# High-risk factors for massive haemorrhage in medical abortion patients with missed miscarriage

**DOI:** 10.1186/s12884-024-06682-x

**Published:** 2024-09-09

**Authors:** Yuru Tan, Sen Li, Hong Xu, Shuying Wang

**Affiliations:** 1 Department of Family Planning, School of Medicine, International Peace Maternity and Child Health Hospital, Shanghai Jiao Tong University, No. 910 Hengshan Road, Xuhui District, Shanghai, China; 2 Department of Gynaecology and Obstetrics, School of Medicine, International Peace Maternity and Child Health Hospital, Shanghai Jiao Tong University, No. 910 Hengshan Road, Xuhui District, Shanghai, China; 3 Shanghai Key Laboratory of Embryo Original Diseases, Shanghai, 200030 China

**Keywords:** Missed miscarriage, Medical abortion, Massive haemorrhage, Emergency surgery

## Abstract

**Background:**

Recently, the incidence of missed miscarriage has gradually increased, and medical abortion is a common method to terminate a pregnancy. In the process of medical abortion, massive vaginal bleeding takes place, leading to emergency surgical haemostasis. Emergency surgery may produce infection and organ damage. Our study aimed to investigate the high-risk factors for massive haemorrhage during a medical abortion.

**Methods:**

A total of 1062 missed miscarriage patients who underwent medical abortion participated in this retrospective study. According to the amount of bleeding, the patients were divided into a massive haemorrhage group and a control group. By comparing the general conditions of the two groups, such as fertility history, uterine surgery history, uterine fibroids, etc., the high-risk factors for massive haemorrhage during medical abortion were identified.

**Results:**

Relative to the control group, the massive haemorrhage group exhibited a higher proportion of patients with a previous artificial abortion (51.9% vs. 38.1%, *P* = 0.001). Additionally, the massive haemorrhage group had a lower percentage of first-time pregnant women (32.1% vs. 40.4%) and a higher proportion of women with shorter pregnancy intervals (44.9% vs. 33.1%, *P* = 0.03). Furthermore, there were notable differences between the two groups regarding maximum fibroid size, the duration of amenorrhea, and gestational week (*P* < 0.05).

**Conclusion:**

In this study, we determined that a history of artificial abortion and an amenorrhea duration of > 11 weeks represented high-risk factors for massive vaginal bleeding during medical abortion in missed miscarriage patients.

## Background

A missed miscarriage is defined as the demise of the embryo, with the retention of all pregnant tissues within the uterus [1]. Its aetiology is attributed to foetal chromosomal abnormalities [2, 3], maternal infection [4], immune system disorders [5], endocrine abnormalities [6], and male factors [7]. In recent years, the incidence of missed miscarriage has gradually increased [8]. Currently, mifepristone and misoprostol are commonly used to terminate missed miscarriage [9]. Medical abortion is a safe and effective method to terminate pregnancy, however, patients undergoing medical abortion may experience substantial vaginal bleeding, requiring emergency surgical haemostasis. Emergency surgery carries the risk of infection, organ damage, and other complications with potential physical and mental consequences. To mitigate the associated risks and complications of emergency surgery, we should identify the high-risk patients prone to massive haemorrhage and strive to reduce the need for emergency surgical procedures.

Currently, limited research has been conducted concerning the high-risk factors associated with severe vaginal bleeding during medical abortion. The objective of this study was to analyse the potential high-risk factors for excessive vaginal bleeding in patients undergoing medical abortion for missed miscarriage. We hypothesised that specific characteristics, including fertility history, previous abortion history, gestational week, uterine fibroids size, pregnancy interval, uterine location, history of uterine surgery, and uterine malformations, may be associated with massive haemorrhage during a medical abortion.

## Methods

### Data collection

This retrospective study was performed at the Family Planning Unit of the International Peace Maternity and Child Health Hospital from January 2017 to December 2021. The medical records of 1062 patients who underwent medical abortion due to missed miscarriage were reviewed. Patients who met the inclusion criteria were included in the study.

Our inclusion criteria were as follows: (1) confirmed pregnancy by blood chorionic gonadotropin and ultrasound examination following 5–24 weeks of amenorrhea; (2) at least two ultrasound scans indicated that the embryo’s development has ceased; (3) termination of pregnancy following admission using mifepristone and misoprostol; (4) provision of patient informed consent to medical abortion, with no contraindications to medical abortion; (5) hospitalised patients.

Our exclusion criteria were as follows: (1) patients who underwent curettage under ultrasound monitoring; (2) patients with a previous history of excessive bleeding during induced abortion (≥ 500 mL for vaginal delivery or ≥ 1000 mL for caesarean delivery); (3) patients with a previous history of excessive bleeding after medical abortion (≥ 100 mL of blood loss within 1 h of medical abortion); (4) patients who were allergic to mifepristone or misoprostol; (5) patients with contraindications to medical abortion, including asthma, glaucoma, and hypertensive diseases; (6) patients with comorbid malignancies; and (7) non-hospitalised patients.

Missed miscarriage was defined by the following criteria [10]: (1) a crown-rump length ≥ 7 mm on ultrasonography without a foetal heartbeat; (2) an average gestational sac diameter ≥ 25 mm with no detected embryo in intrauterine pregnancies; (3) no observable yolk sac and an absent embryo and foetal heartbeat after two weeks in intrauterine pregnancies; and (4) a yolk sac but no foetal heartbeat after 11 days in intrauterine pregnancies.

**Fig. 1:**
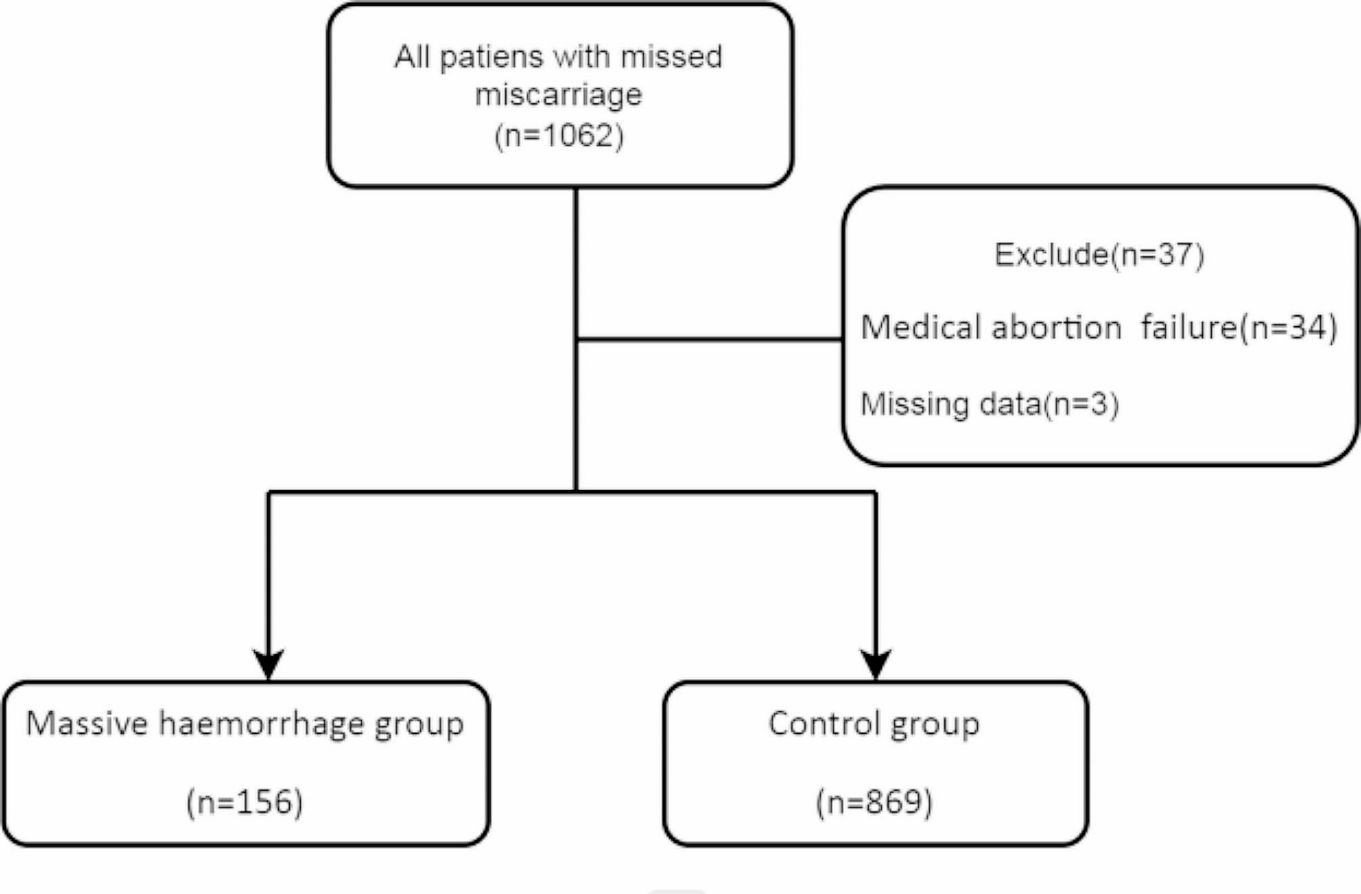
Flowchart of the study workflow

Before performing a medical abortion, exclusion of any contraindications was performed, and patients signed informed consent forms. The standard procedure involved administering mifepristone 100 mg orally once daily for two days. Misoprostol was then administered vaginally within 24 h after the second oral mifepristone dose. When the patient’s gestational age was less than 16 weeks, a single 0.6 mg dose of misoprostol was administered vaginally. However, for patients with a gestational age of 16 weeks or greater, 0.4 mg of misoprostol was administered vaginally every 12 h. The decision to repeat the vaginal administration was founded on the presence of contractions, and this process could be repeated every 12 h for up to three doses. We employed the weighing method to evaluate the amount of vaginal bleeding in patients who underwent abortion. The specific method proceeded as described below. The patient’s vaginal blood was collected on nursing pads, weighed, and the difference between the original weight of the nursing pad and the weight with blood was the patient’s bleeding weight (excluding pregnancy tissue). Bleeding weight divided by the specific gravity of blood 1.05 is the bleeding volume. We avoided the influence of urine on the results when characterising the amount of bleeding. According to Chinese family planning diagnosis and treatment specifications [11], a haemorrhage volume ≥ 200 mL when the gestational age is less than 10 weeks and ≥ 300 mL for patients with a gestational age of 10 weeks or greater were classified as postabortion haemorrhage. It is essential to promptly apply effective haemostatic measures in patients experiencing bleeding during abortion. Given that emergency surgery requires time for preoperative preparation as well as patient transport, it was possible that a patient could continue to bleed during this time. To ensure medical safety by minimising patients bleeding, we developed the following criteria for emergency surgery. The criteria for determining the requirement for emergency surgery were established as cumulative vaginal blood loss of at least 100 mL within 1 h following the medical abortion procedure or a single episode of vaginal bleeding exceeding 80 mL accompanied by active bleeding [12]. All patients meeting the criteria for emergency surgery underwent curettage under ultrasound to remove pregnancy tissue and achieve haemostasis. Of the 1062 patients outlined above, 34 patients were excluded due to medical abortion failure. In addition, three patients with absent blood loss data were also excluded (Fig. 1). Consequently, a total of 1025 patients were included in the study and separated into two groups based on whether they underwent emergency surgery: a massive haemorrhage group and a control group.

Clinical data, encompassing patient age, body mass index (BMI), fertility history, ultrasound results, medical abortion records, surgical records, and other patient outcomes, were obtained and queried through electronic medical records. The variables included in the data collection process included patient age, BMI, the number of pregnancies, history of artificial abortion, history of vaginal delivery, history of caesarean section, history of spontaneous abortion, history of medical abortion, pregnancy interval, fibroids size, the number of fibroids, twin pregnancy, uterine position, adenomyomas, uterine malformations, lactation status, duration of amenorrhea, gestational age, and missed miscarriage duration. Artificial abortion refers to cervical dilation and curettage or vacuum suction to terminate a pregnancy. Gestational age represented the foetal size measured by ultrasound. Amenorrhea refers to the first day of the last menstruation to the day of termination of pregnancy by medical abortion. Missed miscarriage duration refers to the time when the embryo stops developing, denoting the difference between the time of amenorrhea and the actual gestational age of the embryo.

### Data analysis

Statistical product and service solutions (SPSS, version 26.0, www.spss.com) software was employed for data analysis. The massive haemorrhage risk was predicted by considering the raw values of each continuous variable and plotting receiver operating characteristic (ROC) curves. The optimal cut-off value from the ROC curve was utilised to convert the continuous variables into categorical variables (Tables 1 and 2). To evaluate the differences in categorical variables, a chi-square test (χ^2^) was applied. Correlations between categorical variables were examined using column linkage numbers. Multiple linear regression was employed to characterise the presence of collinearity among the variables. Collinearity was defined as a variance inflation factor (VIF) over 10 or a tolerance below 0.1. Logistic regression analysis was performed to determine the crude odds ratio (OR) and corresponding 95% confidence intervals (95% CI). To identify significant independent predictors of massive haemorrhage for missed miscarriage patients who had undergone medical abortion, factors associated with massive haemorrhage were included in the multivariate logistic regression analysis (*P* < 0.2). *P*-values less than 0.05 were deemed statistically significant. The corrplot package was employed to visualise correlations between variables. Forest plots were produced using the forestplot package. All packages were utilised in the R language (version 4.3.2, www.r-project.org).

**Table 1:** Area under the curve (AUC), Maximum Youden Index, and best cut-off values for each variable individually in Predicting the risk of massive haemorrhage

Characteristics	AUC	Youden index	Cut-off value
Age(years)	0.508	0.042	38.5
Amenorrhea duration(weeks)	0.572	0.132	11.5
Gestational weeks(weeks)	0.573	0.103	7.5
Missed miscarriage duration(weeks)	0.529	0.079	3.5
Pregnancy interval(months)	0.469	0.010	1.5

**Table 2:** Comparison of clinical characteristics between the massive haemorrhage Group and the Control Group

Characteristics	Emergency *N* = 156	Controls *N* = 869	χ^2^	*P*-value
Age (years)			2.294	0.130
≤ 38	133(85.3)	777(89.4)		
>38	23(14.7)	92(10.6)		
BMI			2.245	0.523
Underweight	19(12.2)	80(9.2)		
Normal weight	96(61.5)	582(67.0)		
Overweight	33(21.2)	171(19.7)		
Obesity	8(5.1)	36(4.1)		
Pregnancy number			3.760	0.052
1	50(32.1)	350(40.3)		
≥ 2	106(67.9)	519(59.7)		
Vaginal delivery number			1.840	0.175
No	123(78.8)	724(83.3)		
Yes	33(21.2)	145(16.3)		
Cesarean			0.128	0.721
No	130(83.3)	734(84.5)		
Yes	26(16.7)	135(15.5)		
Artificial abortion			10.528	0.001
No	75(48.1)	538(61.9)		
Yes	81(51.9)	331(38.1)		
Medication abortion			0.223	0.637
No	148(94.9)	816(93.9)		
Yes	8(5.1)	53(6.1)		
Spontaneous abortion			0.530	0.467
No	139(89.1)	756(87.0)		
Yes	17(10.9)	113(13.0)		
Pregnancy interval			7.000	0.030
First pregnancy	50(32.1)	351(40.4)		
≤ 36 months	70(44.9)	296(34.1)		
>36 months	36(23.1)	222(25.5)		
Max fibroid size			5.128	0.024
<5 cm	154(98.7)	821(94.5)		
≥ 5 cm	2(1.3)	48(5.5)		
Twin pregnancy			0.071	0.790
No	149(95.5)	834(96.0)		
Yes	7(4.5)	35(4.0)		
Uterine position			4.938	0.085
Anteflexed	135(86.5)	715(82.3)		
Anterior-midline	7(4.5)	25(2.9)		
Retroverted	14(9.0)	129(14.8)		
Uterine malformations			1.476	0.224
No	149(95.5)	807(92.9)		
Yes	7(4.5)	62(7.1)		
Adenomyomas			0.138	0.710
No	155(99.4)	857(98.6)		
Yes	1(0.6)	12(1.4)		
Lactation*			/	0.168
No	154(98.7)	866(99.7)		
Yes	2(1.3)	3(0.3)		
Fibroids number			3.615	0.164
Non	140(89.7)	748(86.2)		
Single	12(7.7)	65(7.5)		
Multiple	4(2.6)	56(6.5)		
Amenorrhea duration (weeks)			9.251	0.002
≤ 11	55(35.3)	421(48.4)		
>11	101(64.7)	448(51.6)		
Gestational weeks (weeks)			6.452	0.011
≤ 7	37(23.7)	296(34.1)		
>7	119(76.3)	573(65.9)		
Missed miscarriage duration (weeks)			3.436	0.064
≤ 3	83(53.2)	531(61.1)		
>3	73(46.8)	338(38.9)		

BMI, body mass index

## Results

Among the cohort of women, a total of 156 missed miscarriages required emergency surgery due to bleeding during medical abortion, while the remaining 869 did not require such intervention. Table 2 outlines the clinical characteristics of the patients in the two groups. Relative to the control group, the massive haemorrhage group exhibited a higher proportion of patients with a previous artificial abortion (51.9% vs. 38.1%, *P* = 0.001). Additionally, the massive haemorrhage group had a lower percentage of first-time pregnant women (32.1% vs. 40.4%) and a higher proportion of women with shorter pregnancy intervals (44.9% vs. 33.1%, *P* = 0.03). Furthermore, there were notable differences between the two groups regarding maximum fibroid size, the duration of amenorrhea, and gestational week (*P* < 0.05). In contrast, there were no noteworthy differences in age, BMI, the number of pregnancies, the number of vaginal deliveries, the number of caesarean deliveries, medical abortions, spontaneous abortions, twin pregnancy, uterine position, uterine malformations, lactation status, adenomyomas, the number of fibroids, or missed miscarriage duration between the two groups (*P* > 0.05).

**Fig. 2:**
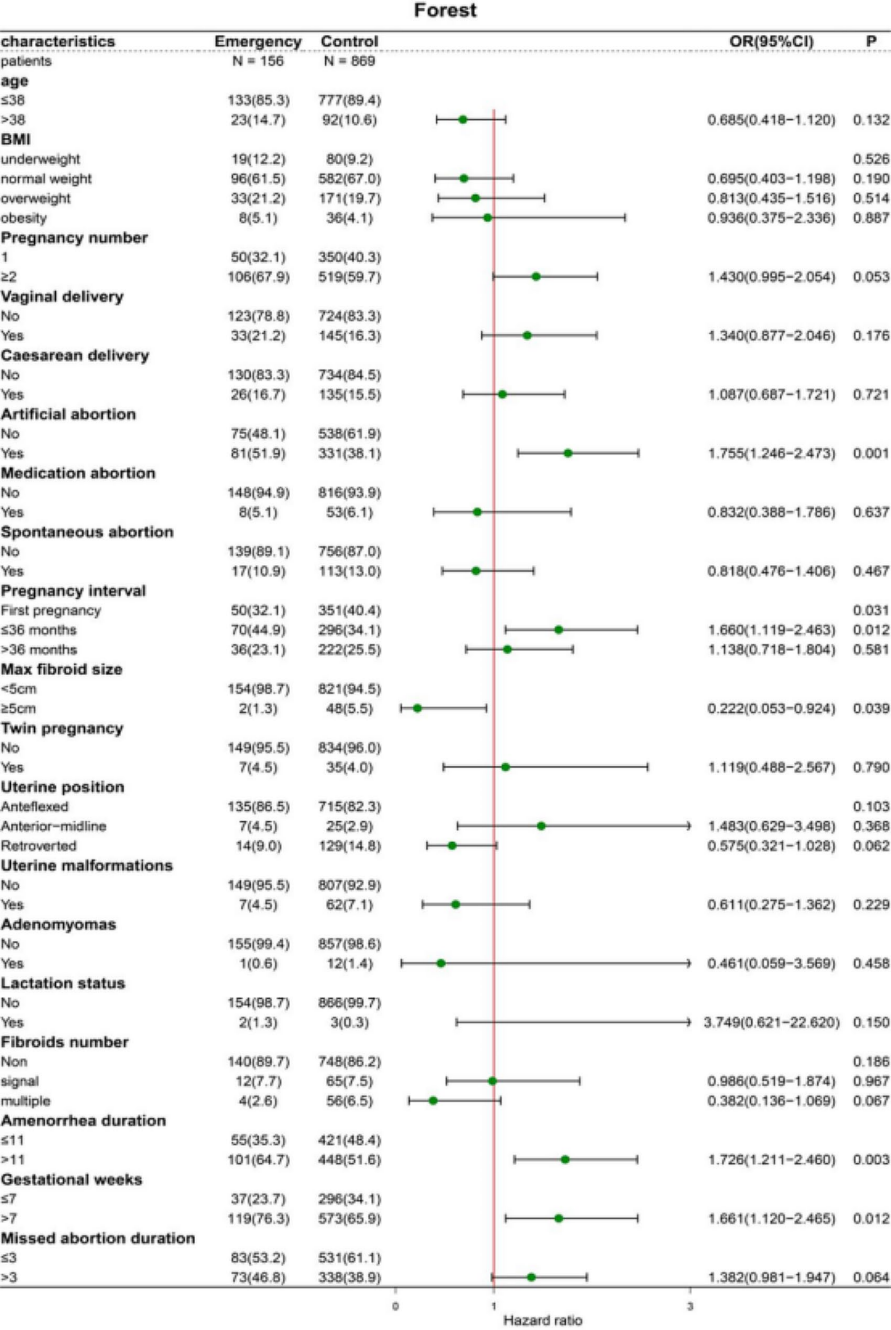
Forest plot depicting univariate analysis results

The findings of the correlation analysis between each categorical variable are presented in Fig. 2. Specifically, a stronger correlation was identified between the pregnancy interval and number of pregnancies. The findings of the univariate regression analysis are outlined in Table 3. Age, the number of vaginal deliveries, the number of artificial abortions, pregnancy interval, the size of the largest fibroid, uterine position, lactation status, the number of fibroids, amenorrhea duration, gestational week, and missed miscarriage duration were significant factors associated with massive haemorrhage (*P* < 0.2). As highlighted in Table 4, collinearity analysis revealed collinearity between gravidity and the pregnancy interval (VIF > 10). The univariate results are presented in a forest plot, found in Fig. 2.

**Table 3:** Outcomes of the univariate regression analysis, specifically focusing on the risk associated with massive haemorrhage

Factors	OR (95%CI)	*P*-value
Age	0.685(0.418–1.120)	0.132
BMI (underweight)		0.526
BMI (normal weight)	0.695(0.403–1.198)	0.190
BMI (overweight)	0.813(0.435–1.516)	0.514
BMI (obesity)	0.936(0.375–2.336)	0.887
Pregnancy number	1.430(0.995–2.054)	0.053
Vaginal delivery number	1.340(0.877–2.046)	0.176
Cesarean number	1.087(0.687–1.721)	0.721
Artificial abortion	1.755(1.246–2.473)	0.001
Medication abortion	0.832(0.388–1.786)	0.637
Spontaneous abortion	0.818(0.476–1.406)	0.467
First pregnancy		0.031
Pregnancy interval ≤ 36 months	1.660(1.119–2.463)	0.012
Pregnancy interval>36months	1.138(0.718–1.804)	0.581
Max fibroid size	0.222(0.053–0.924)	0.039
Twin pregnancy	1.119(0.488–2.567)	0.790
Uterine position(Anteflexed)		0.103
(Anterior-midline)	1.483(0.629–3.498)	0.368
(Retroverted)	0.575(0.321–1.028)	0.062
Uterine malformations	0.611(0.275–1.362)	0.229
Adenomyomas	0.461(0.059–3.569)	0.458
Lactation	3.749(0.621–22.620)	0.150
Fibroids number non		0.186
Single	0.986(0.519–1.874)	0.967
Multiple	0.382(0.136–1.069)	0.067
Amenorrhea duration	1.726(1.211–2.460)	0.003
Gestational weeks	1.661(1.120–2.465)	0.012
Missed miscarriage duration	1.382(0.981–1.947)	0.064

OR, odds ratio; 95%CI, 95% confidence intervals

**Table 4:** Collinearity analysis

Characteristics	Tolerance	VIF
Age	0.891	1.122
Pregnancy number	0.004	249.338
Vaginal delivery number	0.818	1.222
Artificial abortion	0.547	1.827
Pregnancy interval ≤ 36 months	0.004	239.275
Pregnancy interval>36 months	0.005	196.665
Max fibroid size	0.620	1.613
Uterine position: Anterior-midline	0.986	1.014
Uterine position: Retroverted	0.963	1.038
Lactation status	0.971	1.029
Fibroids number: single	0.831	1.204
Fibroids number: multiple	0.646	1.548
Amenorrhea duration (weeks)	0.578	1.729
Gestational weeks (weeks)	0.682	1.466
Missed miscarriage duration (weeks)	0.704	1.421

VIF: variance inflation factor

In the results of the multivariate regression analysis (Table 5), two independent risk factors linked to massive haemorrhage were identified. These factors encompass a history of artificial abortion (OR: 1.82, 95% CI: 1.29–2.57, *P* = 0.001) and an amenorrhea duration > 11 weeks (OR: 1.79, 95% CI: 1.25–2.56, *P* = 0.001).

**Table 5:** Multivariable logistic regression analysis for independent risk factors linked to massive haemorrhage

	OR (95%CI)	*P*-value
Artificial abortion	1.82 (1.29–2.57)	0.001
Amenorrhea duration	1.79 (1.25–2.56)	0.001

OR, odds ratio; 95%CI, 95% confidence intervals

## Discussion

In this study, we investigated the haemorrhage in medical abortion patients with missed miscarriages. The results showed that 15.2% of the patients with missed miscarriage underwent emergency surgical haemostasis due to severe vaginal bleeding during medical abortion. We attempted to identify risk factors associated with this phenomenon, finding that, compared to the control group, the case group had a higher proportion of previous artificial abortions and amenorrhea exceeding 11 weeks. In addition, multivariate logistic regression demonstrated that a combined history of artificial abortion and an amenorrhea duration > 11 weeks were independent risk factors for haemorrhage in missed miscarriage patients.

The definition of post-abortion haemorrhage remains unstandardised. In China, blood volume ≥ 200 mL when the gestational age is less than 10 weeks and ≥ 300 mL for patients with a gestational age of 10 weeks or greater were classified as postabortion haemorrhage [11]. According to the 2013 ACOG guidelines [13], termination of pregnancy between 13 and 26 weeks of gestation is referred to as a second-trimester abortion; massive haemorrhage is defined as the occurrence of clinical reactions like requiring blood transfusion or hospitalisation, or when the amount of postabortion bleeding exceeds 500 mL. According to the time of pregnancy termination, the haemorrhage rate of artificial abortion ranges from 0.3/1000 to 10/1000 [14], and the haemorrhage rate for medical abortion varies between 1% and 15% [15, 16]. In our hospital, missed miscarriage patients lacking high-risk factors and/or gestational age below 10 weeks typically underwent curettage at the outpatient clinic. Termination of pregnancy in the hospital is recommended for missed miscarriage patients beyond 10 weeks or gestational age less than 10 weeks, along with high-risk factors including uterine malformations, five or more prior miscarriages, uterine fibroids larger than 5 cm [17], and recent history of artificial abortion within the last three months. Compared to a prior investigation by Chu [18], the bleeding rate of medical abortion in our study is higher. The potential reason is that the inclusion criteria of this study encompass the above-mentioned hospitalised patients with high-risk factors, whereas Chu counts the bleeding rate of all medical abortion patients, without differentiating whether abortion patients are high-risk groups.

Artificial abortion is a treatment for unplanned pregnancies resulting from contraceptive failure. According to the research report, approximately 60 million induced abortion procedures are performed annually worldwide [19]. In our study, we found that a combined history of artificial abortion can increase the risk of massive haemorrhage during medical abortion. We speculate that the mechanism of this phenomenon is as follows. Artificial abortion is an invasive procedure accompanied by diverse risks and complications, including intrauterine adhesions [20], uterine perforation [21], and secondary infertility [22]. Furthermore, this surgery can inflict varying degrees of harm to the functional and basal layers of the endometrium, negatively impacting endometrial integrity and making a patient prone to endometritis, leading to a thin endometrium [23]. Consequently, during subsequent pregnancies, villus implantation can penetrate deeper into the endometrial layer and reach the myometrium, giving rise to placental adhesions or implantations [24]. During a medical abortion, uterine contractions lead to the gradual separation of the embryo from the implantation site [25, 26], and the local sinusoids open, making a patient more likely to experience postpartum haemorrhage. Therefore, individuals with a history of artificial abortion who undergo medical abortion face a higher risk of significant vaginal bleeding during medical abortion relative to those without a similar history. However, further research is needed to investigate the specific mechanisms involved.

Our research also found that a long amenorrhea duration can also increase the risk of massive haemorrhage. This is consistent with other research [27, 28]. We made the assumptions due to a lack of explanation regarding the corresponding reasons, necessitating further research. The longer the duration of amenorrhea is, the larger the size of the embryo or gestational sac and the more progressive the development of the placenta. This increases the risk of incomplete expulsion of the gestational sac, placenta, and decidua from the uterus during medical abortion, impacting uterine contractions and causing a higher likelihood of vaginal bleeding. In missed miscarriage, where the foetus has stopped developing, there may be a longer period of foetal tissue organisation, causing dense adherence to the endometrium. This can make it more challenging to separate the foetus from the uterine lining during an abortion, resulting in haemorrhage. Additionally, prolonged missed miscarriage can be complicated by coagulopathy, which increases the risk of massive vaginal bleeding during a medical abortion, potentially requiring emergency surgery.

Furthermore, we found that patients with massive haemorrhage have shorter intervals between pregnancies, mirroring the research results of Ekin et al. [29], who found that, compared to the control group, postpartum haemorrhage patients had a higher proportion of interpregnancy intervals. This may be associated with the recovery and damage of the uterus. In previous clinical practice, we identified that lactating pregnant patients were more likely to develop massive vaginal bleeding during medical abortion. A possible explanation for this occurrence is that the hormone fluctuations during lactation can cause a softer uterine texture, weakened contractile function, and increased susceptibility to bleeding. Upon scrutinising our analysis, we found that lactation status was not significantly correlated with the need for emergency surgery. However, this finding may be attributed to the limited number of lactating patients in our study. This requires further studies with large samples across multiple centres.

In addition, we identified an interesting phenomenon in which the proportion of the largest myoma in the bleeding group exceeding 5 cm is smaller. In the bleeding group, the largest myoma diameter is 10.5 cm, while in the control group, the largest myoma is 8.5 cm. Moreover, according to the univariate logistic regression results in this study, the size of the largest fibroid was found to be a protective factor against emergency surgery (Table 3). This finding contradicts previous views on the matter somewhat [30, 31]. The presence of uterine fibroids can impact the contractile function of the uterus, causing bleeding during medical abortion. Additionally, the location of uterine fibroids also governs their influence. More specifically, fibroids located in the intermural space have a greater impact on uterine contraction [32], whereas subserosal fibroids have a reduced effect. However, it is essential to note that in this study, we did not perform further analysis on the location or number of uterine fibroids. This may account for the disparity in the results, necessitating further investigation.

Termination of pregnancy using medicine is a commonly employed approach known as medical abortion, however, it is important to acknowledge that complications can occur during this process. Some of these complications include ineffective termination, incomplete medical abortion, and excessive vaginal bleeding [33]. For patients experiencing significant vaginal bleeding, prompt surgical haemostasis is necessary. Such emergency surgeries, conducted without sufficient preoperative preparation, heighten the risk of anaesthetic complications [34] and increase the likelihood of further complications such as infections [35] and organ damage. Patients often endure significant pain and suffering from suboptimal experiences during these emergency procedures, causing both physical and psychological distress. When dealing with patients seeking medical abortion to terminate their pregnancy, a comprehensive assessment is required. Patients with high-risk factors should make preoperative plans and establish effective communication. For instance, engaging in thorough preoperative discussions to inform patients about potential risks is essential. Additionally, it is necessary to be prepared for emergency cases involving significant vaginal bleeding and take appropriate measures such as preparing for blood transfusion, opening vein access, and early administration of oxytocin, methyl ergonovine and other agents. These actions aim to limit risks and complications linked to emergency procedures, improve patient satisfaction, and protect women’s physical and mental health. We are the first to examine the high-risk factors of massive haemorrhage during a medical abortion in a missed miscarriage. Our study includes numerous high-risk factors potentially associated with vaginal bleeding, which can guide future clinical practice. However, this is a retrospective study with many shortcomings, and a multicentre study with a larger sample size is necessary to obtain more definitive results. Meanwhile, residual confounding by unmeasured variables is still possible, which could have biased our results. For example, in other uterine surgery history (such as cervical surgery, myomectomy and hysteroscopic surgery), the use of anticoagulants may affect haemorrhage. Prospective population studies taking into account more possible confounders are required to investigate the risk of haemorrhage in medical abortion patients with missed miscarriages.

## Conclusion

An integrated history of prior artificial abortion and an amenorrhea duration > 11 weeks are significant independent risk factors for massive vaginal bleeding during medical abortion in missed miscarriage patients undergoing emergency surgery. Age, the number of vaginal deliveries, pregnancy interval, the size of the largest fibroid, uterine position, lactation status, the number of fibroids, gestational week, and missed miscarriage duration were associated with massive haemorrhage. An adequate preoperative plan for high-risk patients can enhance the prognosis of medical abortion.

## Data Availability

No datasets were generated or analysed during the current study.

## Abbreviations

BMI: Body mass index
ROC: Receiver operating characteristic
VIF: Variance inflation factor
OR: Odds ratio
95%CI: 95%confidence intervals
